# 4′-(2-Meth­oxy­phen­yl)-2,2′:6′,2′′-terpyridine

**DOI:** 10.1107/S241431462401143X

**Published:** 2024-11-28

**Authors:** Eric M. Njogu, David O. Juma, Sizwe J. Zamisa, Bernard Omondi, Vincent O. Nyamori

**Affiliations:** a Multimedia University of Kenya, PO Box 15653-00503, Nairobi, Kenya; b School of Chemistry and Physics, University of KwaZulu Natal, Private Bag X54001, Westville, Durban, 4000, South Africa; University of Aberdeen, United Kingdom

**Keywords:** crystal structure, terpyridine

## Abstract

In the title compound, C_22_H_17_N_3_O, the N atoms of the pyridine rings exhibit a typical *trans*–*trans* arrangement: the dihedral angles between the central pyridine ring and the peripheral rings are 22.24 (4) and 2.38 (4)°. In the crystal, pairwise C—H⋯N hydrogen bonds form inversion dimers described by an *R*^2^_2_(6) graph set descriptor, which further inter­act through C—H⋯π and π–π inter­actions, creating a two-dimensional supra­molecular network propagating in the *bc* plane.

## Abstract 2

In the title compound, the dihedral angles between the central pyridine ring and the peripheral rings are 22.24 (4) and 2.38 (4)°. In the crystal, pairwise C—H⋯N hydrogen bonds form inversion dimers described by an *R*^2^_2_(6) graph set descriptor, which further inter­act through C—H⋯π and π–π inter­actions, creating a two-dimensional supra­molecular network propagating in the *bc* plane.

## Graphical abstract

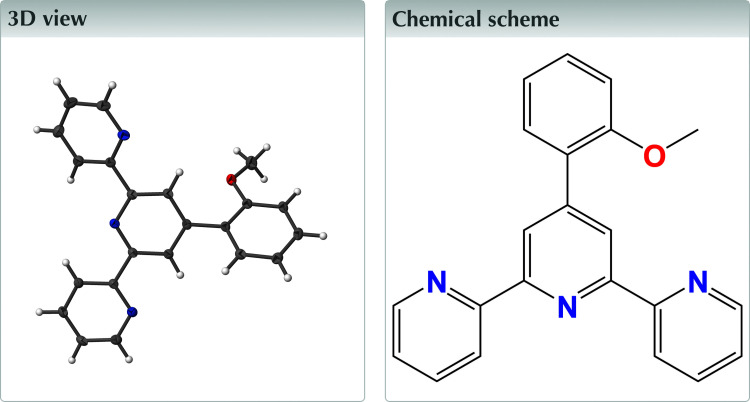

## Structure description

Terpyridines are *N*,*N*,*N*-type pincer ligands that provide tight chelation with various metal cations in a nearly planar *cis*–*cis* geometry of their pyridine N atoms (Wei *et al.*, 2019 ▸). This conformation allows for a good conjugation between the aromatic rings and the metal cation making terpyridine a ‘non-innocent’ ligand, capable of stabilizing low-valency metal ions (García–Domínguez *et al.*, 2017 ▸). The ligand exhibits two possible coordination modes: mono-terpyridine pincer complexes and bis-terpyridine complexes depending on the number of coordinating terpyridine ligands (Taniya *et al.*, 2021 ▸). The transition-metal complexes of 4′-aryl-substituted-2,2′:6′,2"-terpyridines possess rich supra­molecular chemistry (Wei *et al.*, 2019 ▸) as well as biological, DNA binding, and electrochemical properties, which render them as useful candidates for applications in the ﬁelds of medicine and mol­ecular biology (Lazić *et al.*, 2016 ▸). The substituent groups on the ligands may be used to tailor the properties of the resulting coordination complexes (Shi *et al.*, 2006 ▸).

**Figure 1:**
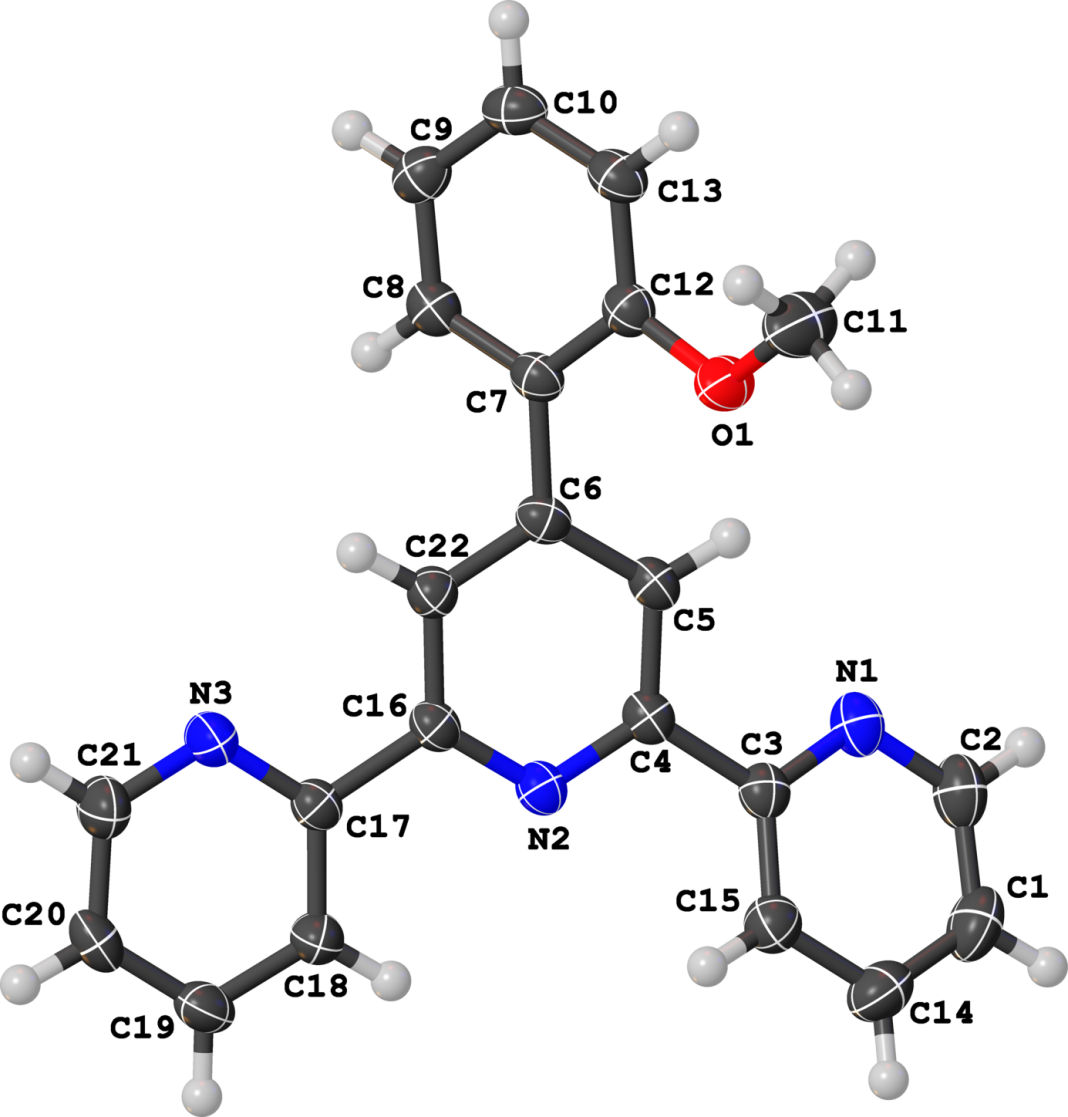
The mol­ecular structure of (**I**) showing displacement ellipsoids drawn at the 50% probability level.

The title compound, C_22_H_17_N_3_O (**I**), is a terpyridine derivative with a 2-meth­oxy­phenyl substituent at the third carbon atom of the central pyridine ring. The crystal structure of the compound contains one mol­ecule in the asymmetric unit in space group *P*2_1_/*c*. As is typical for a non-coordinated terpyridine, the structure exhibits a *trans–trans* arrangement of the pyridine N atoms [N1—C3—C4—N2 = 158.59 (10)°; N3—C17—C16—N2 = −179.09 (10)°], as illustrated in Fig. 1 ▸. The peripheral pyridine rings subtend dihedral angles of 22.24 (4)° (N1 ring) and 2.38 (4)° (N3 ring) with the central pyridine ring. The meth­oxy­phenyl ring is significantly twisted away from the central pyridine ring, with a dihedral angle of 48.93 (4)°. All other geometrical parameters for (**I**) are comparable with those of 4′-(2,4-di­meth­oxy­phen­yl)-2,2′:6′,2′′-terpyridine (Cambridge Structural Database refcode: JEYHED; Demircioğlu *et al.*, 2018 ▸).

**Figure 2:**
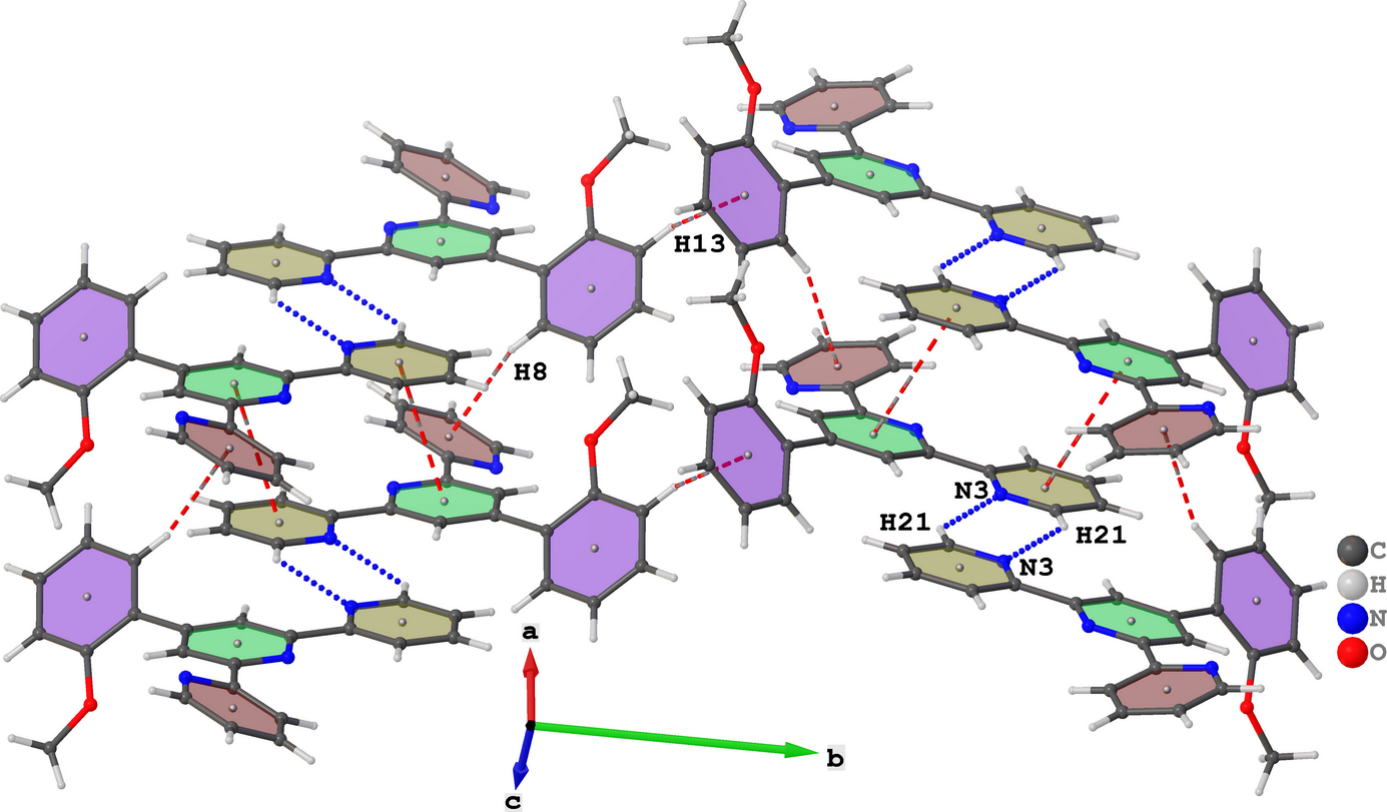
Illustration of inter­molecular C—H⋯N inter­actions in the extended structure of (**I**) depicted as blue dotted lines and C—H⋯π and π–π inter­actions represented by red dashed lines.

**Figure 3:**
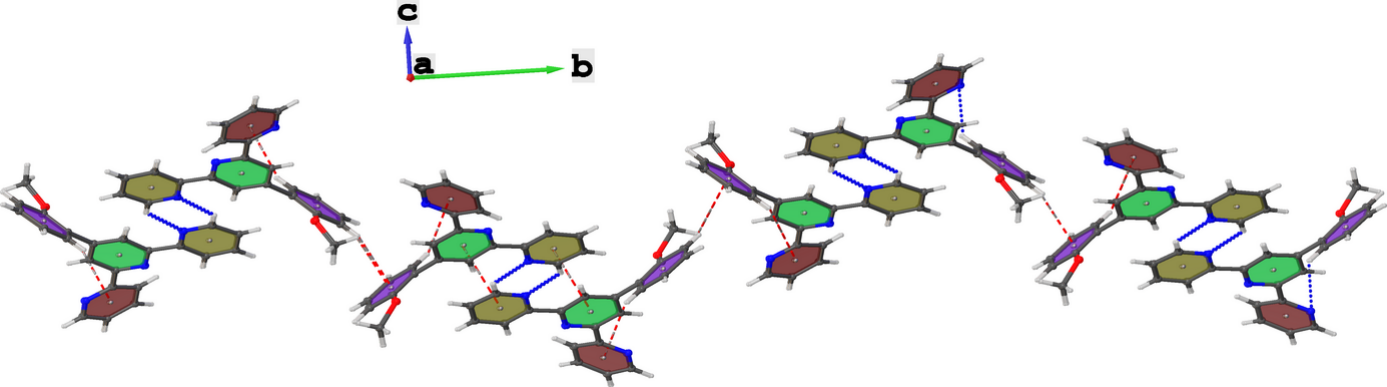
Representation of zigzag propagation patterns of the hydrogen bonded dimers along the crystallographic *b*-axis direction.

In the extended structure of (**I**), weak C—H⋯N hydrogen bonds connect the mol­ecules (Fig. 2 ▸, Table 1 ▸). These inter­actions form hydrogen-bonded cyclic dimers, described by an 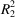
(6) graph set descriptor. The hydrogen-bonded dimers inter­act through C—H⋯π inter­actions, where C8—H8 inter­acts with the centroid of one of the peripheral pyridine rings and C13—H13 inter­acts with the centroid of the meth­oxy-substituted ring. These inter­actions link neighbouring dimers along the *b*-axis direction forming a zigzag pattern, as shown in Fig. 3 ▸. The planarity of the mol­ecules facilitates π–π inter­actions between the central pyridine ring and the other pyridine ring not involved in the C—H⋯π inter­molecular inter­action, with the shortest centroid–centroid separation being 3.5864 (6) Å. The C—H⋯π and π–π inter­actions combine to form a two-dimensional supra­molecular arrangement extending over the crystallographic *bc* plane.

## Synthesis and crystallization

The title compound was synthesized using a method modified from Winter *et al.* (2006 ▸): 2-meth­oxy­benzaldehyde (10 mmol) was dissolved in ethanol (30 ml), cooled to 0 °C, and treated with a 2-acetyl­pyridine/NaOH solution. After stirring for 2 h at 0 °C, 25% aqueous ammonia (30 ml) was added, and the reaction was stirred at room temperature for 18 h. The precipitate was filtered, washed with water and 1:1 water–ethanol, dried under vacuum, and recrystallized from methanol solution to yield X-ray-quality crystals.

## Refinement

Crystallographic data and structure refinement details are summarized in Table 2 ▸.

## Supplementary Material

Crystal structure: contains datablock(s) I. DOI: 10.1107/S241431462401143X/hb4497sup1.cif

Structure factors: contains datablock(s) I. DOI: 10.1107/S241431462401143X/hb4497Isup2.hkl

Supporting information file. DOI: 10.1107/S241431462401143X/hb4497Isup3.cml

CCDC reference: 2405021

Additional supporting information:  crystallographic information; 3D view; checkCIF report

## Figures and Tables

**Table 1:** Hydrogen-bond geometry (Å, °) *Cg*1 and *Cg*4 are the centroids of the N1/C1–3/C14/C15 and C7–C12 rings, respectively.

*D*—H⋯*A*	*D*—H	H⋯*A*	*D*⋯*A*	*D*—H⋯*A*
C21—H21⋯N3^i^	0.95	2.66	3.4138 (16)	137
C8—H8⋯*Cg*1^ii^	0.95	2.68	3.5698 (12)	155
C13—H13⋯*Cg*4^iii^	0.95	2.77	3.5182 (12)	136

Symmetry codes: (i) 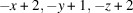; (ii) 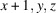; (iii) 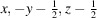.

**Table 2:** Experimental details

Crystal data	
Chemical formula	C_22_H_17_N_3_O
*M*_r_	339.38
Crystal system, space group	Monoclinic, *P*2_1_/*c*
Temperature (K)	100
*a*, *b*, *c* (Å)	7.7366 (3), 29.5787 (10), 7.3852 (3)
β (°)	91.962 (2)
*V* (Å^3^)	1689.03 (11)
*Z*	4
Radiation type	Mo *K*α
μ (mm^−1^)	0.08
Crystal size (mm)	0.43 × 0.28 × 0.26

Data collection	
Diffractometer	Bruker *SMARTAPEX2* area detector
Absorption correction	Multi-scan (*SADABS*; Krause *et al.*, 2015 ▸)
*T*_min_, *T*_max_	0.955, 0.988
No. of measured, independent and observed [*I* > 2σ(*I*)] reflections	36956, 4227, 3728
*R*_int_	0.024
(sin θ/λ)_max_ (Å^−1^)	0.670

Refinement	
*R*[*F*^2^ > 2σ(*F*^2^)], *wR*(*F*^2^), *S*	0.041, 0.119, 1.03
No. of reflections	4227
No. of parameters	236
H-atom treatment	H-atom parameters constrained
Δρ_max_, Δρ_min_ (e Å^−3^)	0.37, −0.24

Computer programs: *APEX2* and *SAINT* (Bruker, 2010 ▸), *SHELXS* (Sheldrick, 2008 ▸), *SHELXL2019/3* (Sheldrick, 2015 ▸) and *OLEX2* (Dolomanov *et al.*, 2009 ▸).

## full crystallographic data

### 4'-(2-Methoxyphenyl)-2,2':6',2''-terpyridine Crystal data

**Table:**

C_22_H_17_N_3_O	*F*(000) = 712
*M**_r_* = 339.38	*D*_x_ = 1.335 Mg m^−^^3^
Monoclinic, *P*2_1_/*c*	Mo *K*α radiation, λ = 0.71073 Å
*a* = 7.7366 (3) Å	Cell parameters from 9871 reflections
*b* = 29.5787 (10) Å	θ = 2.6–28.4°
*c* = 7.3852 (3) Å	µ = 0.08 mm^−^^1^
β = 91.962 (2)°	*T* = 100 K
*V* = 1689.03 (11) Å^3^	Block, yellow
*Z* = 4	0.43 × 0.28 × 0.26 mm

### 4'-(2-Methoxyphenyl)-2,2':6',2''-terpyridine Data collection

**Table:**

Bruker SMART APEX2 area detector diffractometer	4227 independent reflections
Radiation source: microfocus sealed X-ray tube, Incoatec Iµs	3728 reflections with *I* > 2σ(*I*)
Graphite monochromator	*R*_int_ = 0.024
Detector resolution: 7.9 pixels mm^-1^	θ_max_ = 28.4°, θ_min_ = 2.6°
ω and φ scans	*h* = −10→10
Absorption correction: multi-scan (SADABS; Krause *et al.*, 2015)	*k* = −39→39
*T*_min_ = 0.955, *T*_max_ = 0.988	*l* = −9→9
36956 measured reflections	

### 4'-(2-Methoxyphenyl)-2,2':6',2''-terpyridine Refinement

**Table:**

Refinement on *F*^2^	Primary atom site location: structure-invariant direct methods
Least-squares matrix: full	Hydrogen site location: inferred from neighbouring sites
*R*[*F*^2^ > 2σ(*F*^2^)] = 0.041	H-atom parameters constrained
*wR*(*F*^2^) = 0.119	*w* = 1/[σ^2^(*F*_o_^2^) + (0.0697*P*)^2^ + 0.611*P*] where *P* = (*F*_o_^2^ + 2*F*_c_^2^)/3
*S* = 1.03	(Δ/σ)_max_ = 0.001
4227 reflections	Δρ_max_ = 0.37 e Å^−^^3^
236 parameters	Δρ_min_ = −0.24 e Å^−^^3^
0 restraints	

### 4'-(2-Methoxyphenyl)-2,2':6',2''-terpyridine Special details

**Table:**

Geometry. All esds (except the esd in the dihedral angle between two l.s. planes) are estimated using the full covariance matrix. The cell esds are taken into account individually in the estimation of esds in distances, angles and torsion angles; correlations between esds in cell parameters are only used when they are defined by crystal symmetry. An approximate (isotropic) treatment of cell esds is used for estimating esds involving l.s. planes.

### 4'-(2-Methoxyphenyl)-2,2':6',2''-terpyridine Fractional atomic coordinates and isotropic or equivalent isotropic displacement parameters (Å^2^)

**Table:**

	*x*	*y*	*z*	*U*_iso_*/*U*_eq_	
O1	0.47285 (11)	0.30543 (3)	1.08916 (12)	0.02170 (19)	
N1	0.14529 (13)	0.35669 (3)	0.52813 (13)	0.0196 (2)	
N2	0.41380 (12)	0.44519 (3)	0.71159 (13)	0.01598 (19)	
N3	0.78678 (13)	0.50369 (3)	0.89892 (14)	0.0217 (2)	
C1	−0.12572 (16)	0.38731 (5)	0.41546 (18)	0.0264 (3)	
H1	−0.231198	0.382780	0.348239	0.032*	
C2	−0.00699 (16)	0.35250 (4)	0.43771 (17)	0.0235 (2)	
H2	−0.035260	0.323951	0.385741	0.028*	
C3	0.18315 (14)	0.39749 (4)	0.59943 (15)	0.0167 (2)	
C4	0.35441 (14)	0.40258 (3)	0.69551 (14)	0.0158 (2)	
C5	0.44371 (14)	0.36519 (3)	0.76556 (15)	0.0166 (2)	
H5	0.397404	0.335618	0.750921	0.020*	
C6	0.60216 (14)	0.37190 (3)	0.85748 (14)	0.0156 (2)	
C7	0.70993 (14)	0.33454 (3)	0.93456 (14)	0.0158 (2)	
C8	0.88534 (14)	0.33331 (4)	0.89734 (15)	0.0181 (2)	
H8	0.932059	0.356180	0.823112	0.022*	
C9	0.99367 (15)	0.29944 (4)	0.96611 (16)	0.0196 (2)	
H9	1.112894	0.299176	0.939317	0.024*	
C10	0.92535 (15)	0.26616 (4)	1.07407 (16)	0.0199 (2)	
H10	0.997718	0.242459	1.119075	0.024*	
C11	0.41225 (17)	0.27917 (5)	1.23632 (19)	0.0307 (3)	
H11A	0.407983	0.247215	1.201421	0.046*	
H11B	0.296187	0.289352	1.266515	0.046*	
H11C	0.490993	0.282929	1.342035	0.046*	
C12	0.64395 (14)	0.30124 (3)	1.04914 (15)	0.0169 (2)	
C13	0.75168 (15)	0.26701 (4)	1.11749 (15)	0.0188 (2)	
H13	0.706583	0.244292	1.193593	0.023*	
C14	−0.08713 (16)	0.42885 (4)	0.49347 (18)	0.0252 (3)	
H14	−0.166839	0.453225	0.483221	0.030*	
C15	0.07000 (15)	0.43416 (4)	0.58670 (16)	0.0206 (2)	
H15	0.100200	0.462304	0.641067	0.025*	
C16	0.56678 (14)	0.45144 (3)	0.79773 (14)	0.0153 (2)	
C17	0.63187 (14)	0.49871 (3)	0.81402 (14)	0.0155 (2)	
C18	0.53607 (15)	0.53500 (4)	0.74648 (16)	0.0196 (2)	
H18	0.427971	0.530273	0.684452	0.024*	
C19	0.60155 (15)	0.57845 (4)	0.77145 (17)	0.0209 (2)	
H19	0.538544	0.603934	0.727027	0.025*	
C20	0.75910 (15)	0.58400 (4)	0.86153 (16)	0.0199 (2)	
H20	0.805831	0.613325	0.882233	0.024*	
C21	0.84764 (16)	0.54583 (4)	0.92113 (18)	0.0238 (3)	
H21	0.957322	0.549703	0.980925	0.029*	
C22	0.66411 (14)	0.41587 (4)	0.87251 (15)	0.0164 (2)	
H22	0.772010	0.421713	0.933282	0.020*	

### 4'-(2-Methoxyphenyl)-2,2':6',2''-terpyridine Atomic displacement parameters (Å^2^)

**Table:**

	*U*^11^	*U*^22^	*U*^33^	*U*^12^	*U*^13^	*U*^23^
O1	0.0192 (4)	0.0219 (4)	0.0242 (4)	0.0007 (3)	0.0036 (3)	0.0082 (3)
N1	0.0222 (5)	0.0204 (5)	0.0163 (5)	−0.0044 (3)	0.0005 (4)	−0.0007 (3)
N2	0.0178 (4)	0.0157 (4)	0.0146 (4)	−0.0014 (3)	0.0013 (3)	0.0004 (3)
N3	0.0207 (5)	0.0164 (4)	0.0276 (5)	−0.0017 (3)	−0.0043 (4)	0.0011 (4)
C1	0.0196 (6)	0.0363 (7)	0.0230 (6)	−0.0054 (5)	−0.0042 (4)	0.0016 (5)
C2	0.0249 (6)	0.0264 (6)	0.0193 (6)	−0.0081 (4)	−0.0002 (4)	−0.0026 (4)
C3	0.0179 (5)	0.0180 (5)	0.0140 (5)	−0.0023 (4)	0.0008 (4)	0.0019 (4)
C4	0.0182 (5)	0.0156 (5)	0.0139 (5)	−0.0010 (4)	0.0015 (4)	−0.0004 (4)
C5	0.0196 (5)	0.0138 (5)	0.0163 (5)	−0.0016 (4)	0.0010 (4)	−0.0003 (4)
C6	0.0183 (5)	0.0144 (5)	0.0144 (5)	0.0012 (4)	0.0023 (4)	0.0008 (4)
C7	0.0195 (5)	0.0131 (4)	0.0147 (5)	0.0001 (4)	−0.0011 (4)	−0.0008 (4)
C8	0.0207 (5)	0.0169 (5)	0.0168 (5)	−0.0014 (4)	0.0008 (4)	−0.0001 (4)
C9	0.0185 (5)	0.0217 (5)	0.0186 (5)	0.0017 (4)	−0.0011 (4)	−0.0029 (4)
C10	0.0240 (5)	0.0178 (5)	0.0176 (5)	0.0041 (4)	−0.0035 (4)	−0.0014 (4)
C11	0.0263 (6)	0.0348 (7)	0.0315 (7)	0.0028 (5)	0.0078 (5)	0.0155 (5)
C12	0.0186 (5)	0.0162 (5)	0.0158 (5)	−0.0009 (4)	−0.0003 (4)	−0.0011 (4)
C13	0.0249 (5)	0.0157 (5)	0.0156 (5)	−0.0002 (4)	−0.0015 (4)	0.0021 (4)
C14	0.0200 (6)	0.0298 (6)	0.0255 (6)	0.0028 (4)	−0.0014 (5)	0.0035 (5)
C15	0.0205 (5)	0.0207 (5)	0.0205 (6)	−0.0007 (4)	−0.0007 (4)	0.0009 (4)
C16	0.0176 (5)	0.0139 (5)	0.0144 (5)	−0.0008 (4)	0.0020 (4)	0.0007 (4)
C17	0.0173 (5)	0.0148 (5)	0.0144 (5)	−0.0007 (4)	0.0018 (4)	0.0006 (4)
C18	0.0185 (5)	0.0169 (5)	0.0232 (6)	−0.0007 (4)	−0.0020 (4)	0.0029 (4)
C19	0.0231 (6)	0.0153 (5)	0.0246 (6)	0.0013 (4)	0.0020 (4)	0.0035 (4)
C20	0.0255 (6)	0.0147 (5)	0.0199 (6)	−0.0046 (4)	0.0043 (4)	−0.0008 (4)
C21	0.0226 (6)	0.0207 (6)	0.0277 (6)	−0.0043 (4)	−0.0054 (5)	0.0004 (4)
C22	0.0165 (5)	0.0161 (5)	0.0167 (5)	−0.0009 (4)	−0.0002 (4)	0.0010 (4)

### 4'-(2-Methoxyphenyl)-2,2':6',2''-terpyridine Geometric parameters (Å, º)

**Table:**

O1—C11	1.4279 (14)	C9—H9	0.9500
O1—C12	1.3718 (14)	C9—C10	1.3832 (17)
N1—C2	1.3400 (15)	C10—H10	0.9500
N1—C3	1.3449 (14)	C10—C13	1.3922 (16)
N2—C4	1.3454 (13)	C11—H11A	0.9800
N2—C16	1.3372 (14)	C11—H11B	0.9800
N3—C17	1.3415 (14)	C11—H11C	0.9800
N3—C21	1.3403 (14)	C12—C13	1.3949 (15)
C1—H1	0.9500	C13—H13	0.9500
C1—C2	1.3858 (18)	C14—H14	0.9500
C1—C14	1.3855 (18)	C14—C15	1.3854 (16)
C2—H2	0.9500	C15—H15	0.9500
C3—C4	1.4891 (15)	C16—C17	1.4896 (14)
C3—C15	1.3949 (15)	C16—C22	1.3965 (14)
C4—C5	1.3942 (15)	C17—C18	1.3874 (15)
C5—H5	0.9500	C18—H18	0.9500
C5—C6	1.3950 (15)	C18—C19	1.3915 (15)
C6—C7	1.4858 (14)	C19—H19	0.9500
C6—C22	1.3893 (14)	C19—C20	1.3783 (16)
C7—C8	1.3943 (15)	C20—H20	0.9500
C7—C12	1.4060 (15)	C20—C21	1.3848 (16)
C8—H8	0.9500	C21—H21	0.9500
C8—C9	1.3909 (15)	C22—H22	0.9500
			
C12—O1—C11	117.34 (9)	H11A—C11—H11B	109.5
C2—N1—C3	117.01 (10)	H11A—C11—H11C	109.5
C16—N2—C4	117.74 (9)	H11B—C11—H11C	109.5
C21—N3—C17	117.64 (10)	O1—C12—C7	116.06 (9)
C2—C1—H1	120.8	O1—C12—C13	123.82 (10)
C14—C1—H1	120.8	C13—C12—C7	120.11 (10)
C14—C1—C2	118.50 (11)	C10—C13—C12	119.95 (10)
N1—C2—C1	123.94 (11)	C10—C13—H13	120.0
N1—C2—H2	118.0	C12—C13—H13	120.0
C1—C2—H2	118.0	C1—C14—H14	120.7
N1—C3—C4	117.10 (9)	C15—C14—C1	118.69 (11)
N1—C3—C15	122.92 (10)	C15—C14—H14	120.7
C15—C3—C4	119.97 (10)	C3—C15—H15	120.5
N2—C4—C3	115.62 (9)	C14—C15—C3	118.91 (11)
N2—C4—C5	123.16 (10)	C14—C15—H15	120.5
C5—C4—C3	121.21 (9)	N2—C16—C17	117.35 (9)
C4—C5—H5	120.5	N2—C16—C22	122.72 (9)
C4—C5—C6	118.93 (9)	C22—C16—C17	119.93 (10)
C6—C5—H5	120.5	N3—C17—C16	115.79 (9)
C5—C6—C7	123.57 (9)	N3—C17—C18	122.73 (10)
C22—C6—C5	117.84 (9)	C18—C17—C16	121.48 (10)
C22—C6—C7	118.56 (9)	C17—C18—H18	120.7
C8—C7—C6	118.76 (9)	C17—C18—C19	118.64 (10)
C8—C7—C12	118.43 (10)	C19—C18—H18	120.7
C12—C7—C6	122.76 (10)	C18—C19—H19	120.5
C7—C8—H8	119.2	C20—C19—C18	119.08 (10)
C9—C8—C7	121.69 (10)	C20—C19—H19	120.5
C9—C8—H8	119.2	C19—C20—H20	120.8
C8—C9—H9	120.5	C19—C20—C21	118.42 (10)
C10—C9—C8	119.06 (11)	C21—C20—H20	120.8
C10—C9—H9	120.5	N3—C21—C20	123.46 (11)
C9—C10—H10	119.6	N3—C21—H21	118.3
C9—C10—C13	120.71 (10)	C20—C21—H21	118.3
C13—C10—H10	119.6	C6—C22—C16	119.59 (10)
O1—C11—H11A	109.5	C6—C22—H22	120.2
O1—C11—H11B	109.5	C16—C22—H22	120.2
O1—C11—H11C	109.5		
			
O1—C12—C13—C10	−178.02 (10)	C7—C8—C9—C10	0.04 (17)
N1—C3—C4—N2	158.59 (10)	C7—C12—C13—C10	0.84 (16)
N1—C3—C4—C5	−22.36 (15)	C8—C7—C12—O1	176.60 (9)
N1—C3—C15—C14	−1.38 (17)	C8—C7—C12—C13	−2.34 (16)
N2—C4—C5—C6	0.20 (17)	C8—C9—C10—C13	−1.61 (17)
N2—C16—C17—N3	−179.09 (10)	C9—C10—C13—C12	1.18 (17)
N2—C16—C17—C18	1.50 (16)	C11—O1—C12—C7	−165.58 (11)
N2—C16—C22—C6	0.33 (16)	C11—O1—C12—C13	13.32 (16)
N3—C17—C18—C19	−1.50 (18)	C12—C7—C8—C9	1.92 (16)
C1—C14—C15—C3	−0.31 (18)	C14—C1—C2—N1	−1.13 (19)
C2—N1—C3—C4	−178.33 (10)	C15—C3—C4—N2	−21.48 (15)
C2—N1—C3—C15	1.75 (16)	C15—C3—C4—C5	157.57 (11)
C2—C1—C14—C15	1.48 (18)	C16—N2—C4—C3	179.67 (9)
C3—N1—C2—C1	−0.47 (17)	C16—N2—C4—C5	0.65 (16)
C3—C4—C5—C6	−178.77 (10)	C16—C17—C18—C19	177.86 (10)
C4—N2—C16—C17	179.69 (9)	C17—N3—C21—C20	0.10 (19)
C4—N2—C16—C22	−0.92 (16)	C17—C16—C22—C6	179.71 (10)
C4—C3—C15—C14	178.70 (10)	C17—C18—C19—C20	0.23 (17)
C4—C5—C6—C7	−178.80 (10)	C18—C19—C20—C21	1.09 (17)
C4—C5—C6—C22	−0.78 (16)	C19—C20—C21—N3	−1.31 (19)
C5—C6—C7—C8	131.01 (11)	C21—N3—C17—C16	−178.07 (10)
C5—C6—C7—C12	−51.41 (16)	C21—N3—C17—C18	1.33 (17)
C5—C6—C22—C16	0.53 (16)	C22—C6—C7—C8	−46.99 (14)
C6—C7—C8—C9	179.61 (10)	C22—C6—C7—C12	130.59 (11)
C6—C7—C12—O1	−0.99 (15)	C22—C16—C17—N3	1.50 (15)
C6—C7—C12—C13	−179.93 (10)	C22—C16—C17—C18	−177.91 (10)
C7—C6—C22—C16	178.65 (10)		

### 4'-(2-Methoxyphenyl)-2,2':6',2''-terpyridine Hydrogen-bond geometry (Å, º)

*Cg*1 and *Cg*4 are the centroids of the N1/C1–3/C14/C15 and C7–C12 rings, respectively.

**Table:**

*D*—H···*A*	*D*—H	H···*A*	*D*···*A*	*D*—H···*A*
C21—H21···N3^i^	0.95	2.66	3.4138 (16)	137
C8—H8···*Cg*1^ii^	0.95	2.68	3.5698 (12)	155
C13—H13···*Cg*4^iii^	0.95	2.77	3.5182 (12)	136

Symmetry codes: (i) −*x*+2, −*y*+1, −*z*+2; (ii) *x*+1, *y*, *z*; (iii) *x*, −*y*−1/2, *z*−1/2.
